# St. Nicholas

**Published:** 1874-02

**Authors:** 


ST. NICHOLAS, is the title of the largest, best and the freshest Magazine for boys and girls, in the land. Filled with handsome illustrations; running over with choice, instructive and amusing articles; stories and poetry; it is just such a magazine as will fascinate and captivate the young. Parents can confer no greater pleasure for so small an outlay upon their children, than to place *St. Nicholas* in their hands. Address Scribner & Co., New York. Price $3.00 per annum.

